# Active Human Complement Reduces the Zika Virus Load via Formation of the Membrane-Attack Complex

**DOI:** 10.3389/fimmu.2018.02177

**Published:** 2018-10-17

**Authors:** Britta Schiela, Sarah Bernklau, Zahra Malekshahi, Daniela Deutschmann, Iris Koske, Zoltan Banki, Nicole M. Thielens, Reinhard Würzner, Cornelia Speth, Guenter Weiss, Karin Stiasny, Eike Steinmann, Heribert Stoiber

**Affiliations:** ^1^Division of Virology, Medical University of Innsbruck, Innsbruck, Austria; ^2^CNRS, CEA, IBS, University of Grenoble Alpes, Grenoble, France; ^3^Division of Hygiene and Medical Microbiology, Medical University of Innsbruck, Innsbruck, Austria; ^4^Department of Internal Medicine II, Medical University of Innsbruck, Innsbruck, Austria; ^5^Center for Virology, Medical University of Vienna, Vienna, Austria; ^6^Department of Molecular and Medical Virology, Ruhr University Bochum, Bochum, Germany

**Keywords:** complement, Zika virus, C1q, IgM, MAC, lysis

## Abstract

Although neglected in the past, the interest on Zika virus (ZIKV) raised dramatically in the last several years. The rapid spread of the virus in Latin America and the association of the infection with microcephaly in newborns or Guillain-Barré Syndrome in adults prompted the WHO to declare the ZIKV epidemic to be an international public health emergency in 2016. As the virus gained only limited attention in the past, investigations on interactions of ZIKV with human complement are limited. This prompted us to investigate the stability of the virus to human complement. At low serum concentrations (10%) which refers to complement concentrations found on mucosal surfaces, the virus was relatively stable at 37°C, while at high complement levels (50% serum concentration) ZIKV titers were dramatically reduced, although the virus remained infectious for about 4–5 min under these conditions. The classical pathway was identified as the main actor of complement activation driven by IgM antibodies. In addition, direct binding of C1q to both envelope and NS1 proteins was observed. Formation of the MAC on the viral surface and thus complement-mediated lysis and not opsonization seems to be essential for the reduction of viral titers.

## Introduction

Within the genus *Flavivirus* (family *Flaviviridae*), several members were identified as human pathogens including dengue (DEN), Japanese encephalitis (JE), tick-born encephalitis (TBE), West-Nile, (WN), yellow fever (YF) and Zika (ZIK) viruses (1). The main route of infections is mediated by arthropods such as mosquitoes or ticks (2, 3). The viruses contain a single stranded RNA of positive polarity with a size of about 11 kb. The RNA is translated as a polyprotein and cleaved by viral and host-encoded proteases into seven non-structural (NS) and three structural proteins, including the capsid, membrane (prM/M) and envelope (E) protein (3). Interestingly, two flavivirus proteins are characterized as main participants in interactions with the immune system. The E protein binds to the cell surface and mediates fusion after endocytic virus uptake. The majority of the neutralizing antibody responses is directed against the E protein. NS1 functions as regulator of viral transcription and has been shown to antagonize the anti-viral immune response by interfering with the interferon pathway. In addition, NS1 interacts with several proteins of the complement system (4, 5).

As a first line of the defense mechanism of the innate immune system, complement activation triggers a proteolytic cascade leading to release of anaphylatoxins, chemokines and cytokines, opsonization and phagocytosis of invading pathogens and killing by the formation of the membrane-attack complex (MAC) (6). Upon viral entry, either the classical, the lectin or the alternative pathway is initiated. The classical pathway is activated by binding of C1q to immune complexes consisting of IgG or IgM bound to the viral surfaces or by direct interactions of viral proteins with C1q (7, 8). Carbohydrates expressed on the surface of pathogens or ficolins bound to viral surfaces may be recognized by the mannan-binding lectin (MBL) which triggers the lectin pathway. The alternative pathway is constitutively activated due to spontaneous hydrolysis of C3 and drives the amplification of the classical and the lectin pathways. The three activation pathways merge in cleavage of C3 into C3a and C3b by the C3-convertases and the formation of the C5-convertase. Cleaved and thus activated C5b initiates the lytic pathway. C5b becomes associated with C6, C7, C8 and several C9 proteins, referred to as membrane-attack complex (MAC) responsible for formation of the lytic pore resulting in destruction of viral pathogens (6).

The interest for ZIK virus (ZIKV) raised only recently, due to massive spread of the virus mainly in Latin America (9). Infection may cause severe neurological complications mainly in newborn children of infected mothers during pregnancy (10) and is associated with Guillain-Barré Syndrome in adults (11). As, in contrast to other members of the *Flaviviridae* family, investigations on the interaction of ZIKV with proteins of the complement cascade are limited, the aim of this study was to assess the potential complement activating capacity of ZIKV. A further aim was to identify proteins involved in this putative ZIKV-complement interaction and to investigate whether complement can reduce the viral titer.

## Materials and methods

### Cells and viruses

Aedes albopictus C6/36 mosquito cells were grown in Dulbecco's modified Eagle's medium (DMEM; Invitrogen, Carlsbad, USA) supplemented with 10% heat-inactivated fetal calf serum (FCS), antibiotic-antimycotic solution [10,000 units/mL penicillin, 10,000 μg/mL streptomycin, and 25 μg/mL Amphotericin B], L-glutamine, and non-essential amino acids (Gibco, Dublin Ireland) at 28°C in 5% CO_2_.Alternatively, the human cell line A549 was used and cultivated under the same conditions. ZIKV strain MRS_OPY_Martinique_PaRi_2015 (GenBank: KU647676) was provided by European Virus Archive (Marseille, France). For propagation of ZIKV, cells were seeded in culture plates to get confluence of about 80% at the day of infection. Cells were washed with phosphate-buffered saline (PBS) and ZIKV was added with a multiplicity of infection (MOI) of 0.1. After 1 h at 37°C, cells were washed twice with PBS and fresh medium was added. Depending on the growth kinetics of the cell line, the supernatants were harvested and filtered through a 0.45-μm filter to remove cell debris. To generate high titer viral stocks the supernatants were centrifuged overnight (Rotanta 460R Hettich; 4,600 rpm, 16 h, 4°C). The concentrated supernatants were aliquoted and stored at −80°C. All experiments were performed under bio-safety level-2 conditions.

### Human serum samples

Normal human serum (NHS) was purchased from Dunn Labortechnik GmbH (Ansbach, Germany) and stored in aliquots at −80°C. For experimental procedures, serum was thawed only once and kept on ice. Some aliquots from the serum pool were heat inactivated (hiNHS; 56°C, 30 min) and served as controls. Complement C9-or C1q-depleted human serum and purified C9 or C1q were purchased from CompTech (Tylor, Texas USA).

### Plaque assay

To count plaque-forming units (PFU), the virus [total volume 100 μL/sample] was serially diluted 1:10 and added to Vero cells grown in 6-well or 12-well plates. ZIKV samples were incubated with the cells for 1 h at 37°C and overlaid with plaque agarose (Biozym Scientific GmbH, Hessisch Oldendorf, Germany). Four days after incubation at 37°C, viral plaques were visualized by crystal violet staining. The viral titers were expressed as PFU/mL, calculated as [(number of plaques per well) × (dilution)]/(inoculum volume).

### Serum-sensitivity assay

ZIKV [1 x 10^6^ PFU/mL] was incubated with 10%, 20% or 50% (final concentration) NHS, heat-inactivated NHS (hiNHS), or DMEM (supplemented with FCS) as controls. When indicated, complement-depleted sera were used instead of NHS. To block the lectin pathway, a mixture of sunflower MASP inhibitor (SFMI)-1 (3.2 mM) and SFMI-2 (3.2 mM) peptides was used (Metabion, Planegg, Germany). These peptides are known to selectively inhibit MBL-associated serine protease (MASP)-1 and -2 (12). For some experiments, putative IgM in human serum were blocked by an affinity purified goat IgG against human IgM as recommended by the manufacturer (Bethyl Laboratories, Montgomery, USA). After an incubation time of 1 h at 37°C, all samples were serially diluted and titrated on Vero cells to determine the viral titer by plaque assay. When indicated, ZIKV [1 x 10^6^ PFU/mL] derived from A549 cells was used.

### Inhibition of complement activation

To prevent activation of all complement pathways, ZIKV [1 x 10^6^ PFU/mL] was incubated with 50% NHS in the presence of EDTA [1, 2.5 or 5 mM] or Mg^2+^-EGTA [5 mM] for 1 h at 37°C. Immediately after, the virus-containing samples were serially diluted and titrated on 12-well plates of overnight-plated Vero cells. One h after incubation at 37°C, plaque agarose was overlaid. Four days post infection, the visualization and calculation of PFUs were performed as described for Plaque Assay.

### Inhibition of MAC formation

The contribution of the MAC was determined by using C9-deficient serum as follows: ZIKV (1 x 10^6^ PFU/mL) was incubated for 1 h at 37°C in the presence of active or heat-inactivated C9-depleted human serum. As a control, the depleted serum was reconstituted with purified C9 protein (60 mg/mL). The titration and plaque visualization were performed as described above. In parallel, viral RNA was analyzed as described below.

### C1q binding ELISA

To investigate the interaction of the complement component C1q with ZIKV-derived proteins, 5 μg/mL of recombinant ZIKV envelope or NS1 protein [MyBioSource, San Diego, USA] or 100 μL of ZIKV supernatant derived from C6/36 cells [containing 5 x 10^6^ PFU/well] were coated in carbonate buffer (pH 9.6) on a 96-well flat-bottom plate (Maxisorp, Nunc, Roskilde, Denmark) overnight at 4°C. After washing thrice with 200 μL PBS containing 0.05% Tween 20 (PBS-T), blocking solution (3% non-fat dry milk in PBS) was added and allowed to incubate for 30 min at room temperature. Subsequently, 100 μL of purified C1q protein was added as indicated in the figure and incubated with gentle shaking for 2 h at room temperature. The plates were washed five times with PBS-T, followed by the addition of 50 μL/well of homemade polyclonal rabbit antibody against the globular heads of human C1q (1:500). After 1 h at room temperature, the plates were washed again and 50 μL of a goat anti-rabbit IgG conjugated with horseradish peroxidase (HRP) was added (1:10,000). The plates were again incubated for 1 h at room temperature. For detection of bound antibodies, 200 μL 3,3′,5,5′-tetramethylbenzidine (TMB) solution were added following the manufacturer's instructions. The absorbance was measured at 450 nm, using a Bio-Rad plate reader.

### PCR to determine the relative viral titer

ZIKV [1 × 10^8^ RNA copies/mL] was mixed with 10, 20, or 50% (final concentration) NHS or hiNHS and DMEM (supplemented with FCS) as controls. Additionally, 1 mg/mL RNase A [Macherey-Nagel 740505, Dueren, Germany] was added to digest the RNA of lysed viral particles. The mixture [total volume 100 μL/sample] was incubated for 3 h at 37°C in a thermoshaker. Afterwards, the residual viral RNA of remaining intact virions was harvested using the NucliSENS easyMAG system (BioMérieux, Vienna, Austria) as recommended by the manufacturer. To measure the ZIKV-specific RNA, a reverse transcription-PCR (RT-PCR) was done, using the iScript One-Step RT-PCR kit (Quanta; Biorad, Munich, Germany). Primer and probe sequences as well as the thermal profile of the PCR were performed according to the protocol of Lanciotti et al. (13). The relative reduction of the viral RNA was calculated as follows: *ct*-value of the heat-inactivated serum controls was set at 100%. As a reduction of 3.2 ct-units in the real-time PCR equals 1 log (= 90%), changes of the viral RNA in active serum were determined relative to the heat-inactivated serum controls.

### Statistical analysis

Statistical analysis was performed using GraphPad Prism 7.0 software. All experiments were repeated at least three times in duplicate. The difference between two groups was assessed by *t*-test. When comparing more than two groups, ANOVA followed by Bonferroni *post-hoc* tests was performed. A 95% significance level (*p* < 0.05) was considered statistically significant and indicated in the figures as follows: ^*^p < 0.05 to *p* = 0.01, ^**^p < 0.01 to *p* = 0.001, ^***^p < 0.001 to *p* = 0.0001 and ^****^p < 0.0001.

## Results

### Serum neutralization of ZIKV particles

**Figure 1:**
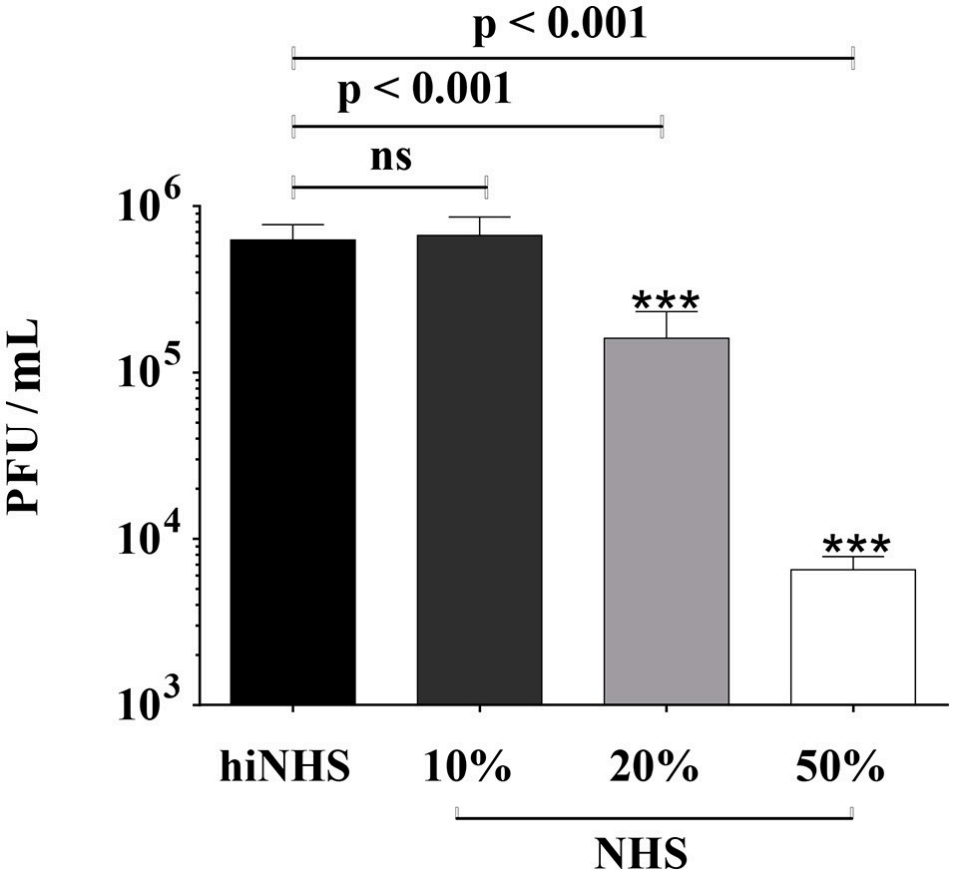
Effect of human serum on ZIKV infectivity. ZIKV [1 x 10^6^ PFU/mL] was incubated with active normal human serum (NHS) or 50% heat-inactivated NHS (hiNHS) for 1 h at 37°C. Thereafter, 10-fold dilutions of pre-incubated virus-serum mixtures were titrated on Vero cells. Plaques were visualized 4 days post infection using crystal violet staining. Data were analyzed with one-way-ANOVA with Bonferroni *post-hoc* comparison (***p < 0.001; ns indicates not significant). Mean data of at least 3 independent experiments are shown. The error bars represent the standard deviation.

To analyze the stability of ZIKV in human serum, insect (mosquito) cell line derived ZIKV was incubated with increasing amounts of pooled NHS and the number of infectious virions was quantified by plaque titration on Vero cells. NHS was tested for the presence of flavivirus antibodies (Supplementary Figure 1) to exclude antibody-mediated viral neutralization. The titer of ZIKV was reduced in a concentration-dependent manner in the presence of NHS, compared to heat-inactivated serum (hiNHS), which was used at a concentration of 50% (Figure 1). The presence of 10% NHS did not affect ZIKV infectivity (Figure 1), whereas 20% NHS yielded a titer reduction of more than 0.5 log. Increasing the serum concentration up to 50% resulted in a viral titer reduction of approximately 2 log (Figure 1). As the plaque counts of the hiNHS control were comparable to the initial viral load [1 x 10^6^ PFU/mL] of the mock medium (data not shown), heat sensitive factors of human serum appear to be involved, implicating the complement system as anti-viral factor for this neutralization.

### Time-dependent stability of ZIKV

**Figure 2:**
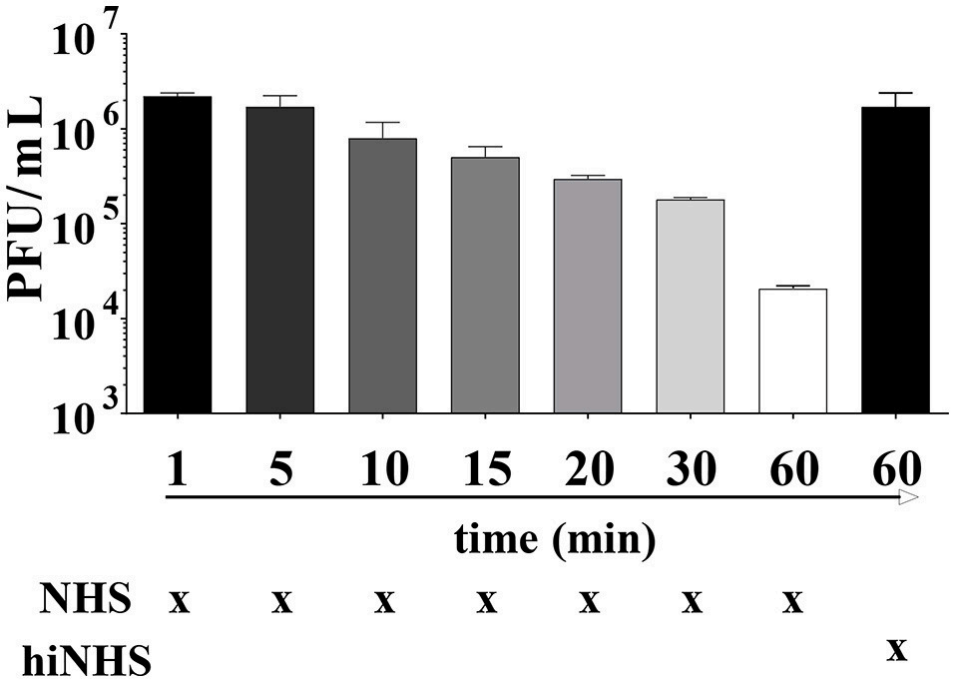
Time as a limiting factor on ZIKV infectivity. ZIKV [1 x 10^6^ PFU/mL] was mixed with 50% active or heat-inactivated NHS and incubated for different time points (ranging from 1 min to 1 h) at 37°C. Virus-containing samples were then serially diluted and titrated on Vero cells. After 1 h incubation at 37°C, the cells were overlaid with agarose. Viral concentration was determined 4 days post infection using crystal violet staining. Mean data of 3 independent experiments are shown. The error bars represent the standard deviation.

The transmission of ZIKV by mosquito species like *Aedes aegypti* occurs at short time intervals (14). Consequently, the time frame between viral inoculation into the skin and receptor-mediated endocytosis by human cells will probably be shorter than the preselected incubation time of 60 min in the serum resistance assays described above. The infectivity of ZIKV remained unaffected for at least 4–5 min. A decrease in plaque titers was observed only after prolongation of the incubation time with about 1 log reduction after 30 min (Figure 2).

### Antiviral activity of human serum is mediated mainly by the classical complement pathway

**Figure 3:**
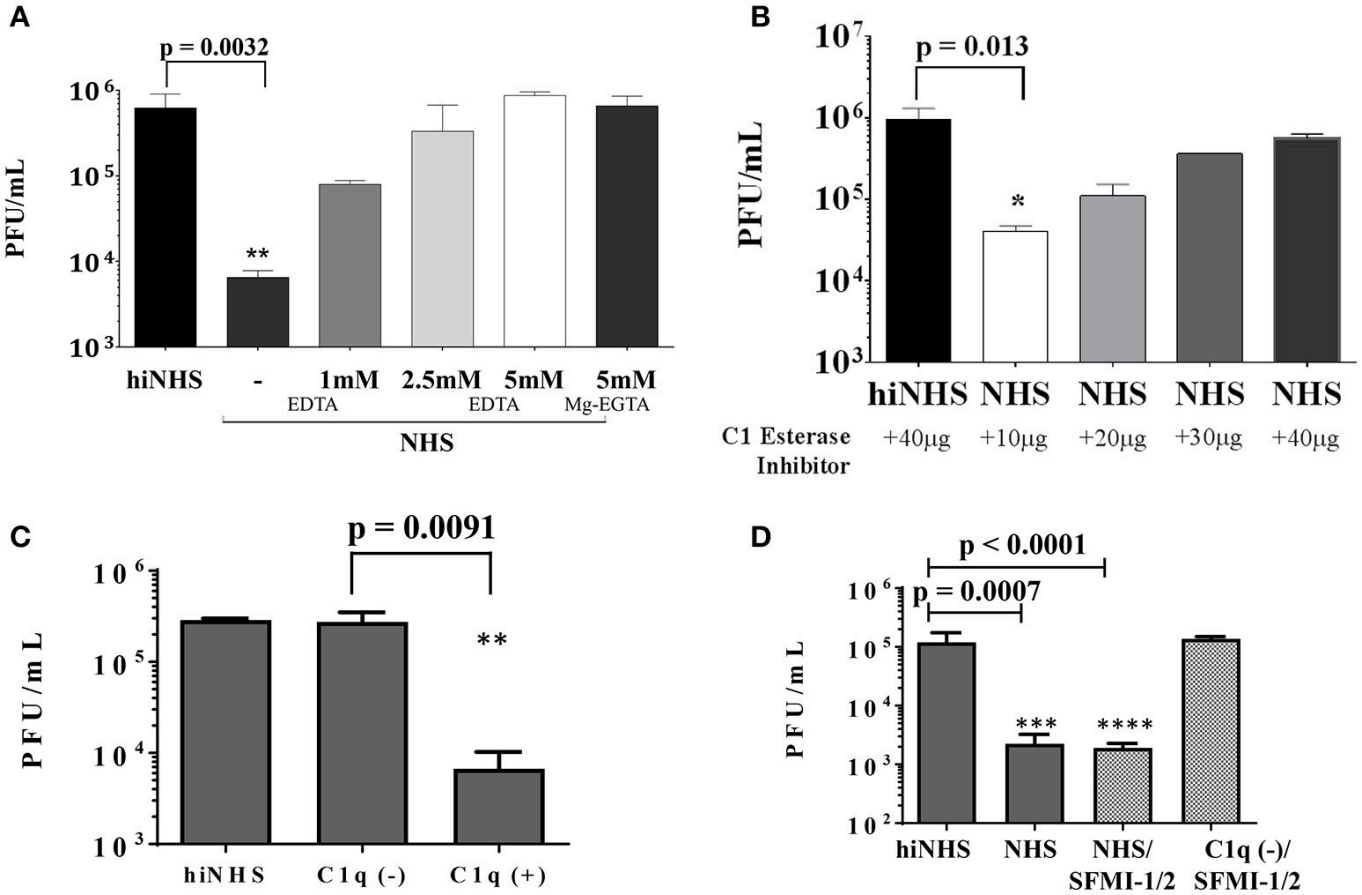
**(A)** EDTA inhibition confirms complement contribution to viral lysis. ZIKV [1 x 10^6^ PFU/mL] was incubated with active or heat-inactivated human serum in the presence of increasing amounts of EDTA or Mg^2+^-EGTA. DMEM (not shown) and hiNHS were used as controls. **(B)** To prevent activation of the classical and lectin complement pathways, active or heat-inactivated human serum was pre-incubated with increasing amounts of C1 esterase inhibitor as indicated. **(C)** In the absence of C1q, most of the virus remained infectious whereas addition of purified C1q (70 μg/mL) restored the lytic effect on the virus. HiNHS was set to 100%. **(D)** Blocking of the lectin pathway by synthetic peptides (SFMI-1/2) did not rescue the virus. Combination of C1q-depleted serum and SFMI-1/2 served as additional control showing that the peptides had no effect on the infectivity of the virus. In all experiments, the viral titer was determined by plaque assays using Vero cells. Data were analyzed with one-way-ANOVA with Bonferroni *post-hoc* comparison (**p < 0.005). All virus lysis experiments were conducted in duplicates and repeated three times. The data represent mean values, and the error bars show standard deviations.

The loss of anti-viral activity of human serum against ZIKV (shown in Figure 1), when hiNHS was used, suggests that a complement-dependent mechanism is involved. To confirm this hypothesis, complement activation of all three pathways was inhibited by adding the Mg^2+^ and Ca^2+^ chelator EDTA, which inhibited complement-mediated lysis of ZIKV in a dose-dependent manner (Figure 3A).

While the initial plaque titer was reduced up to 2 orders of magnitude in 50% NHS, the addition of 5 mM EDTA fully inhibited the ZIKV neutralization activity of NHS. These results indicate that the sensitivity of ZIKV to human serum is mediated by complement activation. We further characterized the participation of the classical and lectin pathways. As both pathways are essentially linked to the availability of Ca^+^ ions, the activation can be efficiently blocked with Mg^2+^-EGTA, whereas the alternative pathway remains active (15). ZIKV was incubated with NHS pretreated with 5 mM Mg^2+^-EGTA (Figure 3A). Like the 5 mM EDTA treatment, Mg^2+^-EGTA fully inhibited the ZIKV neutralization activity of NHS, excluding the participation of the alternative pathway. Therefore, it is likely that the anti-viral activity of human serum is mainly mediated by the classical/lectin pathway of complement. To further investigate the role of the classical/lectin pathway in the complement-mediated viral neutralization, complement activation was inhibited by C1 esterase inhibitor (C1-INH). The serine protease inhibitor C1-INH is a natural regulator of the activation of the classical and lectin pathways. The human serum was pre-incubated with increasing amounts of C1-INH prior to the serum resistance assay of ZIKV. The viral neutralization was inhibited by C1-INH in a concentration-dependent manner, further confirming the involvement of the classical and/or the lectin pathway (Figure 3B). Additionally, C1q-depleted serum was used to discriminate between activation of the classical and lectin pathways. In the absence of C1q, the viral titer was nearly unaffected. In contrast, addition of C1q at a concentration of 70 μg/mL to the depleted serum restored the capacity of the serum to reduce the viral titer (Figure 3C), indicating that the classical pathway is the main trigger for the observed reduction of the viral titer. To further investigate a putative contribution of the lectin pathway, insect-derived ZIKV was incubated with NHS in the presence of the peptides SFMI-1/2. Surprisingly, these MASP-blocking peptides were unable to rescue the viral titer (Figure 3D). By contrast, no lysis was observed when ZIKV was treated with SFMI-1/2 in C1q-depleted serum. These results underscored the dominant role of the classical pathway for the reduction of ZIKV load.

### Neutralization of ZIKV is linked to natural IgM antibodies

**Figure 4:**
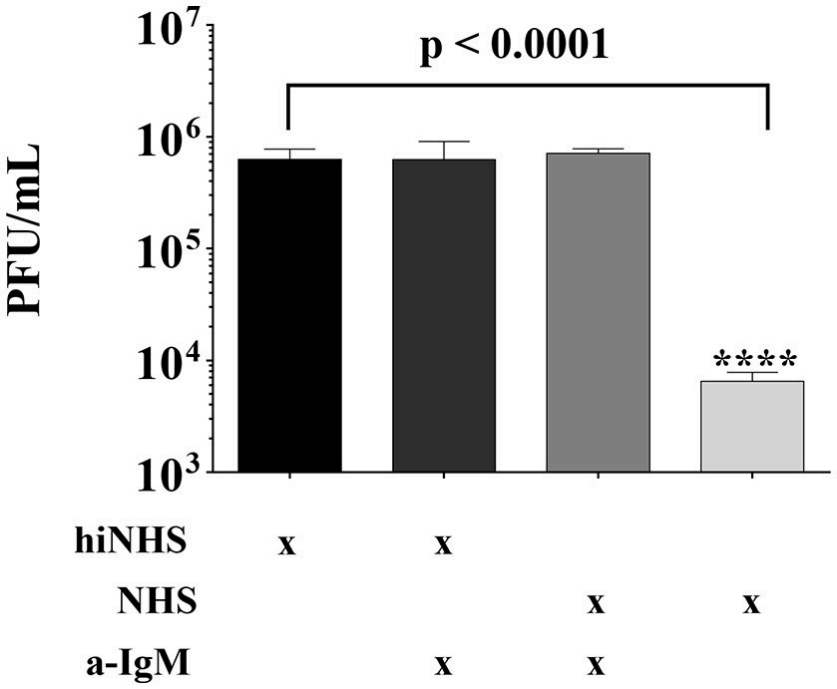
Natural IgM blocking results in ZIKV rescue. Anti-human IgM blocking antibodies were incubated with 50% NHS or hiNHS for 30 min on ice before ZIKV was added. After incubation of 1 h at 37°C, the virus-serum mixture was serially diluted and titrated on Vero cells. After 1 h r incubation at 37°C, the cells were overlaid with agarose. Viral concentration was determined4 days post infection using crystal violet staining. All virus lysis experiments were conducted in triplicate, and the error bars show standard deviations.

As a putative binding of IgM to viral particles may trigger activation of the classical complement pathway (16), active NHS was pre-incubated with anti-IgM antibodies prior to ZIKV addition. As shown before (Figure 1), the titer of ZIKV was reduced at about 2-logs in the presence of 50% (non-treated) NHS, compared to hiNHS. Interestingly, the viral titer was equivalent to the hiNHS control in the presence of IgM-blocking antibodies (Figure 4), indicating that natural IgM antibodies in human serum are involved in complement-mediated neutralization of ZIKV. The presence of insect-specific IgM antibodies in the serum pool was further confirmed by FACS using the insect cell line C6/36, which gave a clear positive signal, when incubated with the serum pool. In contrast, cell lines of human origin, such as A549, remained negative (Supplementary Figure 2).

### C1q binding to ZIKV proteins

**Figure 5:**
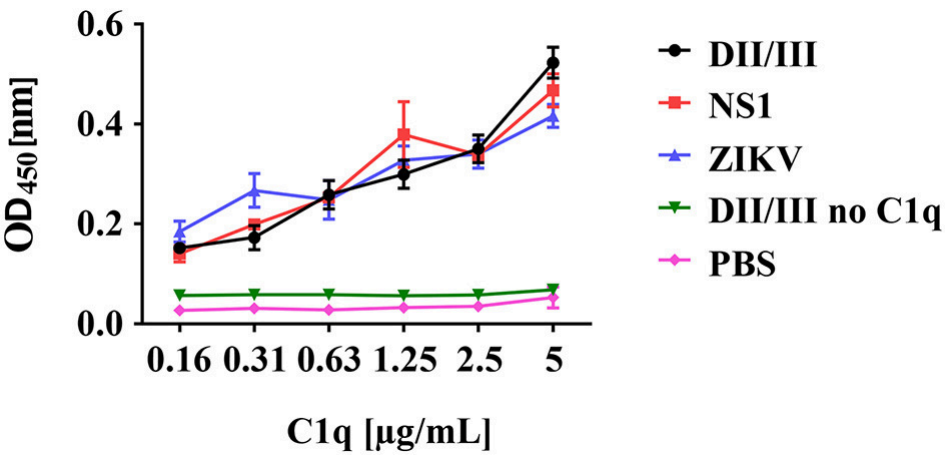
Binding of C1q to recombinant ZIKV envelope (E) and NS1 proteins. ZIKV proteins or viral particles were coated to ELISA plates and incubated with decreasing amounts of C1q as indicated. Bound C1q was detected using a polyclonal anti-C1q antibody followed by a HRP-labeled goat anti-rabbit IgG and visualized by TMB. The absorbance was measured at 450 nm, using a Bio-Rad plate reader. Data show the mean of two experiments performed in duplicate.

For WNV it has been reported that C1q directly interacts with the E protein. Further, the NS1 protein of DENV is known to bind C1q. These observations prompted us to test if the ZIKV E and/or NS1 proteins may interact with C1q. The viral recombinant proteins and entire viral particles were coated in an ELISA plate and incubated with increasing amounts of C1q. As shown in Figure 5, both recombinant viral proteins NS1 and the envelope protein bound C1q in a dose-dependent manner similarly to ZIKV particles, indicating that, beside IgM, a direct activation of the classical pathway is possible.

### Lysis of ZIKV derived from a human cell line

**Figure 6:**
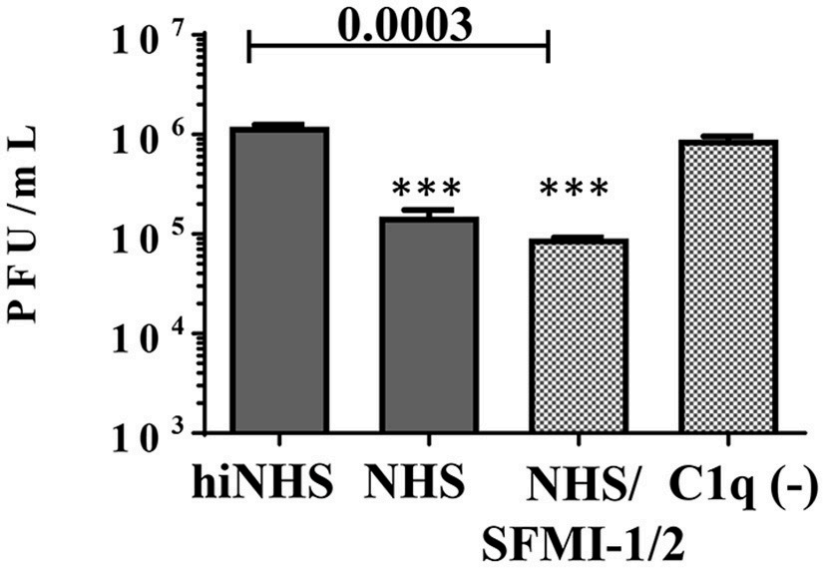
Dissecting the role of the lectin and classical pathways on ZIKV derived from the human cell line A549. NHS (50%) reduced the viral titer for about one order of magnitude. Inhibition of the lectin pathway by a peptide mix of SFMI-1 and 2 had no effect. By contrast, C1q depletion rescued the virus and most of the virus remained infectious. Viral titer was determined by plaque assays using Vero cells. Data were analyzed with one-way-ANOVA with Bonferroni *post-hoc* comparison (**p < 0.005). All virus lysis experiments were conducted in duplicates and repeated two times. The data represent mean values, and the error bars show standard deviations.

As expected, NHS showed no reactivity against human A549 cells (Supplementary Figure 2). Thus, we concluded that a contribution of IgM against ZIKV derived from human cells was unlikely and provided a tool to investigate the significance of C1q binding to the envelope proteins, at least *in vitro*. In addition, the serum was tested negative for the presence of antibodies with cross-reactivity against flaviviruses (Supplementary Figure 1), which excluded a possible trigger of the classical pathway by putative “contaminating” antibodies. As shown in Figure 6, A549-derived ZIKV was sensitive to human serum, although the reduction of the viral titer was less pronounced compared to ZIKV harvested from insect cells. Again, the MASP-blocking peptides were unable to rescue the virus. By contrast, incubation of A549-derived ZIKV with C1q depleted serum had no effect on the viral titer. As NHS was unable to reduce the amount of A549-derived ZIKV in the presence of EGTA or Mg^2+^-EGTA (data not shown), we concluded that a direct binding of C1q to the virus significantly contributes to the control of ZIKV in NHS.

### Complement induces lysis of ZIKV

**Figure 7:**
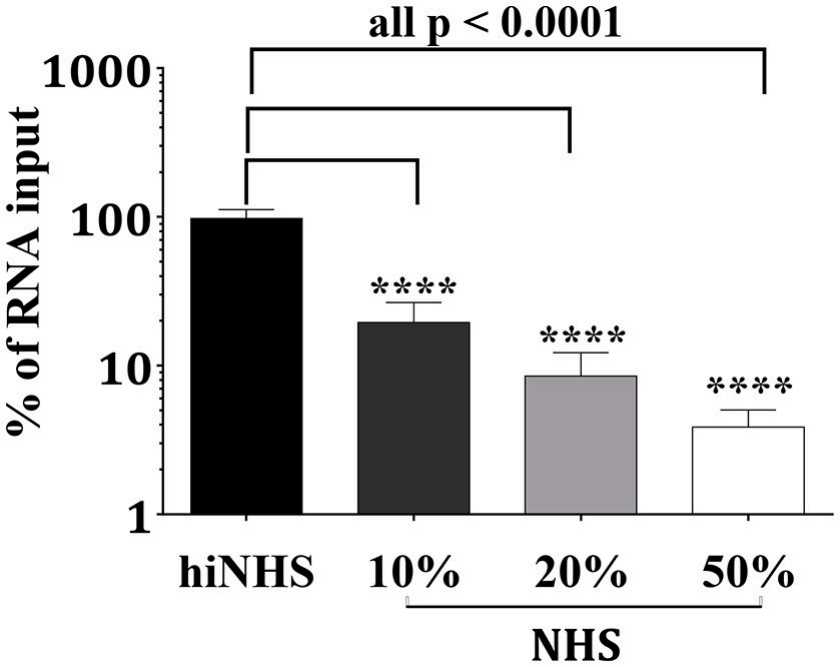
Reduction of viral infectivity is linked to decreased RNA levels. After incubating ZIKV with active or heat-inactivated human serum, the viral RNA was digested by addition of RNases. Three hours after incubation at 37°C, the remaining genomic material was extracted and quantified by RT-PCR. The RNA copy number was calculated from the amount of RNA obtained by incubation of the virions with 50% hiNHS, which was set to 100%. Results are given as % of RNA loss. Data were analyzed with one-way-ANOVA with Bonferroni *post-hoc* comparison (****p < 0.0001). All virus lysis experiments were conducted in triplicate. The data represent mean values, and the error bars show standard deviations.

**Figure 8:**
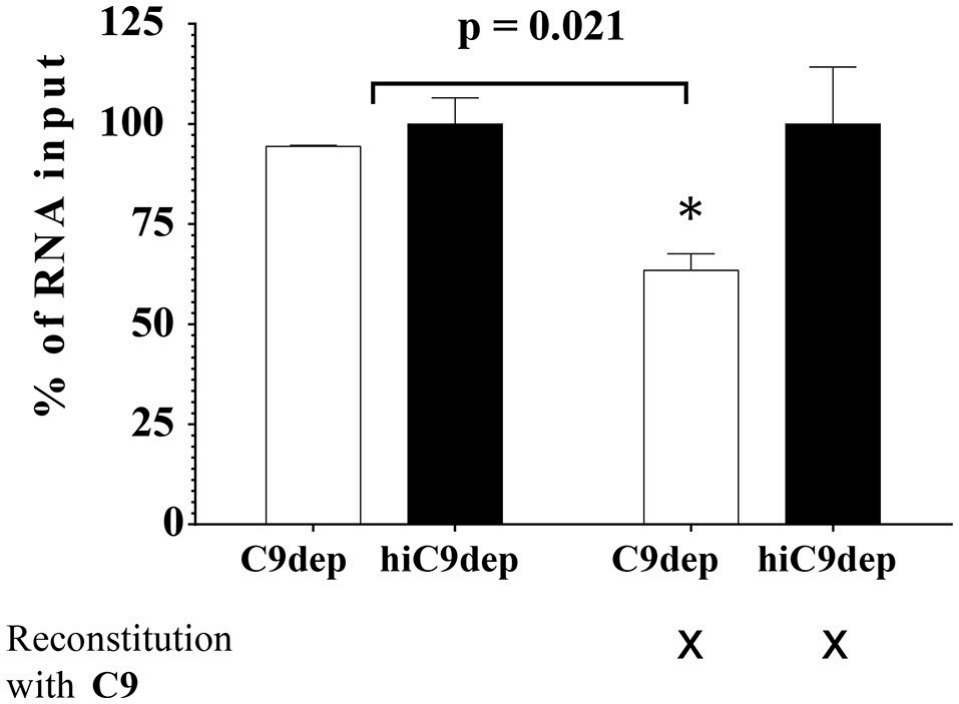
Blocking the assembly of MAC leads to virus rescue. ZIKV [1 x 10^6^ PFU/mL] was exposed to either 50% C9-depleted (C9dep) human serum or 50% heat-inactivated C9-depleted (hiC9dep) serum for 1 h at 37°C. As a control, the depleted serum was reconstituted with purified C9 protein, adjusted to its natural concentration in serum [60 μg/mL]. During incubation, the RNA of lysed ZIKV was digested by external RNase addition. Subsequently, the amount of complement lysis-resistant virions was determined by RT-PCR. The RNA copy number was calculated by incubation of the virions with 50% hiNHS, which was set to 100%. Results are given as % of RNA loss. All assays were performed in triplicate. The data represent mean values, and the error bars show standard deviations.

Complement-mediated neutralization of ZIKV may be due to deposition of proteins such as C3b fragments, which may hide viral epitopes important for infection or, alternatively, may occur by lysis of virions due to formation of the MAC. To analyze whether complement-mediated viral neutralization is linked to MAC assembly, we established an RNase digestion assay followed by PCR. ZIKV was incubated with NHS or hiNHS in the presence of RNases. The formation of the MAC enables the entry of RNases into the virions and digestion of viral RNA. By contrast, intact ZIKV, which are opsonized with C3b, are not affected. Quantification of the virus by real-time PCR (RT-PCR) showed a reduction of RNA copies only when MAC formation occurred. Similar to previous plaque titrations (Figure 1), the presence of human serum induced a concentration-dependent reduction of initial RNA levels (Figure 7). When serum was inactivated, no significant changes in RNA copy number were observed, compared to the virus incubated with mock medium (DMEM, 10% FCS; not shown). To further confirm the importance of MAC formation for the reduction of viral titers, C9-depleted serum was used. ZIKV was exposed to C9-depleted human serum in the presence of RNases. As a control, the C9-depleted serum was reconstituted with purified C9 protein adjusted to its natural concentration in serum (60 μg/mL). As expected, complement-induced lysis was inhibited in the absence of C9 (Figure 8). When the serum was reconstituted with purified C9 protein, a decrease of RNA was observed, indicating that the lytic function of complement was restored. Similarly, C9-depleted serum had no effect in the plaque assay (data not shown). In line with the results obtained by PCR, the neutralization capacity was restored in the plaque assay when purified C9 was re-added to the depleted serum (data not shown).

## Discussion

Our study focused on the anti-viral activity of NHS and identified the complement system as an immediate innate immune response against ZIKV. To mimic the environmental conditions of infection in several compartments, different initial concentrations of complement have been chosen for the *in vitro* experiments. Once a blood-feeding female mosquito transmits the virus, ZIKV is confronted with high concentrations of complement. In contrast, sexual transmission exposes the virus to lower levels of complement at mucosal surfaces. Thus, 10% NHS followed by exposure to 20 and 50% NHS has been used for the *in-vitro* studies. Experiments using Mg^2+^-EGTA showed that the alternative complement pathway is not involved in the reduction of the viral titer. The ZIKV neutralization assays with NHS in the presence of C1-INH indicated that the classical complement pathway is triggered. However, C1-INH may also interfere with the lectin pathway by inhibiting the MASP-1/MASP-2 proteases of the MBL-MASP complexes (17). Therefore, experiments with C1q-depleted serum were performed which clearly indicate that activation of the classical pathway is mainly involved in the reduction of the viral titer. This came by surprise as mosquito cell-derived virus exhibits a mix of high-mannose and paucimannose glycans which should favor binding of MBL (18). In addition, the participation of the lectin pathway to the neutralization of different members of the flaviviruses was shown in former studies. In line with these observations, Fuchs et al. demonstrated direct binding of MBL to WNV, which triggers the activation of MASP-2, followed by opsonization of the virion surface, which finally hampers the viral fusion with host membranes (19). Furthermore, Avirutnan *et al*. confirmed the involvement of MBL, since around 80% of the neutralizing capacity of all DENV serotypes was lost when mouse serum from MBL-A/C^−/−^ mice was used (20). In addition, they showed that MBL, but not C1q or C5, was necessary to neutralize the virus, which states against the participation of the classical complement activation pathway and the formation of membrane attack complex. However, in our setting, blocking of the lectin pathway by synthetic peptides (i.e., SFMI-1 and 2) did not rescue the virus, indicating that the classical pathway is mainly responsible for the reduction of the viral titer.

In line with our observation that ZIKV E interacts with C1q, Douradinha *et al*. reported a direct binding of C1q to recombinant E protein of DENV and viral particles (21). This finding was confirmed by proteomic analysis demonstrating that samples from DENV-infected patients as well as purified domain III of DENV envelope protein bind several complement components among which C1q (22, 23). As in addition, binding of C1q to NS1 of DENV is described (24), we tested the possibility of a direct interaction of C1q with viral particles and purified recombinant NS1 and E proteins of ZIKV. Direct binding of C1q to ZIKV or its recombinant proteins was indeed observed, which prompted us to test if serum immunoglobulins generate immune complexes able to trigger the classical pathway. As we could exclude the presence of cross-reactive IgG from other flaviviruses, we investigated the involvement of natural antibodies. A first hint for the presence of anti-insect IgM was obtained by FACS analysis showing that IgM antibodies in the blood recognized insect cells, whereas cell lines of human origin gave no signal. In addition, ZIKV derived from monkey or human cell lines is more resistant to complement-mediated lysis and complement activation is independent of IgM, further indicating that insect-specific immunoglobulins are involved (manuscript in preparation). This allows the conclusion that neither flavivirus-specific IgG nor IgM is present in the serum pool. Although IgM affinity is low for antigens compared to antibodies of other isotypes (25, 26), it was previously shown that natural IgM antibodies are involved in the elimination of viruses like vesicular stomatitis virus (27, 28), lymphocytic choriomeningitis virus (29) or influenza virus (16, 30–32). In this regard, Beebe and Cooper reported that natural IgM antibodies form virus-immune complexes induced activation of the classical complement pathway. Once initiated at the viral surface, the activation resulted in a massive deposition of C3b, hampering the viral attachment to susceptible host cells (27). As the serum pool used in the present study was tested for the absence of flavivirus-specific antibodies, the natural IgM immunoglobulins are probably directed against insect-like structures such as glycosylation patterns or surface molecules. This is supported by the FACS analysis, which indicated that the serum contained IgM antibodies recognizing insect cells but not cells from human origin. Further, blocking of IgM in the serum pool resulted in complete rescue of the virus. However, these data should be interpreted with caution, as the generated immune complex between IgM and the goat anti-human IgM antibodies may consume complement, which is thus no more available for lysis of the virus. This may also explain, why blocking of IgM by the Ab resulted in a complete rescue of the virus. Our results are in contrast to *in vivo* WNV experiments by Diamond et al., who demonstrated that natural IgM were insufficient to provide protection against WNV (virus derived from C6/36 cells). This may be due to the experimental setting, as mice in animal facilities may have no contact with mosquitos and therefore probably lack IgM against insect antigens (33).

Our data provide evidence that the source of cells used for cultivation of the virus is an important parameter. For lysis induction of ZIKV derived from insect cells, probably both IgM and direct binding of C1q to the virus may account for activation of the classical pathway. By contrast, ZIKV derived from human cells may interact with C1q only. This would explain why the titer of the virus from insect cells is reduced in the order of two magnitudes, while ZIKV derived from human cells is more stable toward complement mediated neutralization.

Opsonization is discussed as a main factor of complement-mediated flavivirus neutralization, as the efficiency of MAC formation may be limited due to the small surface size of the viral particle (5). These results contradict our findings of the effect of C9-depletion on RNA level and in plaque assays, which clearly indicated that the assembly of the MAC was crucial for anti-viral activity by complement-mediated virolysis. Our findings are supported by former *in vivo* studies of WNV suggesting a contribution of all activation pathways (34–36). The dependence on C9 for the reduction of the viral titer is in contrast to previous observations by Mehlhop et al., who highlighted that neither C5-depleted nor C5-deficient human or mouse sera significantly affected antibody-independent neutralization of WNV, indicating a C5-independent mechanism (37). Opsonization of the viral surface by complement proteins, which may cover viral proteins essential for infection, seems to be sufficient to inhibit infection (37). However, opsonization with purified C1q had no effect on the infectivity of the virus (data not shown). Similarly, covering HIV and parainfluenza virus by C3b seems to reduce infectivity of these viruses (38–40). However, due to data obtained with C9-depleted sera, we suggest that opsonization itself is not the major mechanism for the reduction of the viral RNA level and infectious titers of ZIKV in our experiments.

Nevertheless, our results indicate that natural IgM antibodies may have a protective function against ZIKV. Whether these natural IgM protect against infection by other mosquito-transmitted viruses *in vivo* remains to be determined. However, it would be interesting to test whether the mild disease progression observed in many ZIKV infected individuals is partly due to the presence of natural IgM against insect components, which trigger complement activation and thus reduce the viral titer.

## Author contributions

BS, SB, ZM, DD, IK, and ZB experimental work, data interpretation, drafting the article, and final approval. NT, RW, CS, KS, ES, and GW study design, data interpretation, critical revision of the article, and final approval. HS study design, data interpretation, drafting the article, critical revision of the article, and final approval.

### Conflict of interest statement

The authors declare that the research was conducted in the absence of any commercial or financial relationships that could be construed as a potential conflict of interest.

## References

[1] Lindenbach BD Rice CM. Molecular biology of flaviviruses. Adv Virus Res. (2003) 59:23–61. 10.1016/S0065-3527(03)59002-9 14696326

[2] Kuno G Chang GJ. Biological transmission of arboviruses: reexamination of and new insights into components, mechanisms, and unique traits as well as their evolutionary trends. Clin Microbiol Rev. (2005) 18:608–37. 10.1128/CMR.18.4.608-637.2005 16223950 PMC1265912

[3] Hasan SS Sevvana M Kuhn RJ Rossmann MG. Structural biology of Zika virus and other flaviviruses. Nat Struct Mol Biol. (2018) 25:13–20. 10.1038/s41594-017-0010-8 29323278

[4] Avirutnan P Mehlhop E Diamond MS. Complement and its role in protection and pathogenesis of flavivirus infections. Vaccine (2008) 26(Suppl. 8):I100–7. 10.1016/j.vaccine.2008.11.061 19388173 PMC2768071

[5] Conde JN Silva EM Barbosa AS Mohana-Borges R. The Complement system in flavivirus infections. Front Microbiol. (2017) 8:213. 10.3389/fmicb.2017.00213 28261172 PMC5306369

[6] Holers VM. Complement and its receptors: new insights into human disease. Annu Rev Immunol. (2014) 32:433–59. 10.1146/annurev-immunol-032713-120154 24499275

[7] Thielens NM Tacnet-Delorme P Arlaud GJ. Interaction of C1q and mannan-binding lectin with viruses. Immunobiology (2002) 205:563–74. 10.1078/0171-2985-00155 12396016

[8] Huber G Banki Z Lengauer S Stoiber H. Emerging role for complement in HIV infection. Curr Opin HIV AIDS (2011) 6:419–26. 10.1097/COH.0b013e3283495a26 21825871

[9] Oladapo OT Souza JP De Mucio B De Leon RGP Perea W Gulmezoglu AM. WHO interim guidance on pregnancy management in the context of Zika virus infection. Lancet Global Health (2016) 4:E510–1. 10.1016/S2214-109X(16)30098-5 27211476

[10] Johansson MA Mier-Y-Teran-Romero L Reefhuis J Gilboa SM Hills SL. Zika and the Risk of Microcephaly. N Engl J Med. (2016) 375:1–4. 10.1056/NEJMp1605367 27222919 PMC4945401

[11] Cao-Lormeau VM Blake A Mons S Lastere S Roche C Vanhomwegen J. Guillain-Barre Syndrome outbreak associated with Zika virus infection in French Polynesia: a case-control study. Lancet (2016) 387:1531–9. 10.1016/S0140-6736(16)00562-6 26948433 PMC5444521

[12] Kocsis A Kékesi KA Szász R Végh BM Balczer J Dobó J. Selective inhibition of the lectin pathway of complement with phage display selected peptides against mannose-binding lectin-associated serine protease (MASP)-1 and−2: significant contribution of MASP-1 to lectin pathway activation. J Immunol. (2010) 185:4169–78. 10.4049/jimmunol.1001819 20817870

[13] Lanciotti RS Kosoy OL Laven JJ Velez JO Lambert AJ Johnson AJ. Genetic and serologic properties of Zika virus associated with an epidemic, Yap State, Micronesia, (2007). Emerg Infect Dis. (2008) 14:1232–9. 10.3201/eid1408.080287 18680646 PMC2600394

[14] Pingen M Bryden SR Pondeville E Schnettler E Kohl A Merits A. Host inflammatory response to mosquito bites enhances the severity of arbovirus infection. Immunity (2016) 44:1455–69. 10.1016/j.immuni.2016.06.002 27332734 PMC4920956

[15] Des Prez RM Bryan CS Hawiger J Colley DG Function of the classical and alternate pathways of human complement in serum treated with ethylene glycol tetraacetic acid and MgCl2-ethylene glycol tetraacetic acid. Infect Immun. (1975) 11:1235–43. 806523 10.1128/iai.11.6.1235-1243.1975 PMC415205

[16] Jayasekera JP Moseman EA Carroll MC. Natural antibody and complement mediate neutralization of influenza virus in the absence of prior immunity. J Virol. (2007) 81:3487–94. 10.1128/JVI.02128-06 17202212 PMC1866020

[17] Parej K Dobo J Zavodszky P Gal P. The control of the complement lectin pathway activation revisited: both C1-inhibitor and antithrombin are likely physiological inhibitors, while alpha2-macroglobulin is not. Mol Immunol. (2013) 54:415–22. 10.1016/j.molimm.2013.01.009 23399388

[18] Hacker K White L De Silva AM. N-linked glycans on dengue viruses grown in mammalian and insect cells. J Gen Virol. (2009) 90:2097–106. 10.1099/vir.0.012120-0 19494052 PMC2887570

[19] Fuchs A Lin TY Beasley DW Stover CM Schwaeble WJ Pierson TC. Direct complement restriction of flavivirus infection requires glycan recognition by mannose-binding lectin. Cell Host Microbe. (2010) 8:186–95. 10.1016/j.chom.2010.07.007 20709295 PMC2929649

[20] Avirutnan P Hauhart RE Marovich MA Garred P Atkinson JP Diamond MS. Complement-mediated neutralization of dengue virus requires mannose-binding lectin. MBio (2011) 2:e00276-11. 10.1128/mBio.00276-11 22167226 PMC3236064

[21] Douradinha B Mcburney SP Soares De Melo KM Smith AP Krishna NK Barratt-Boyes SM. C1q binding to dengue virus decreases levels of infection and inflammatory molecules transcription in THP-1 cells. Virus Res. (2014) 179:231–4. 10.1016/j.virusres.2013.11.007 24246304 PMC3946857

[22] Fragnoud R Flamand M Reynier F Buchy P Duong V Pachot A Differential proteomic analysis of virus-enriched fractions obtained from plasma pools of patients with dengue fever or severe dengue. BMC Infect Dis. (2015) 15:518 10.1186/s12879-015-1271-7 26572220 PMC4647599

[23] Huerta V Ramos Y Yero A Pupo D Martin D Toledo P. Novel interactions of domain III from the envelope glycoprotein of dengue 2 virus with human plasma proteins. J Proteomics (2016) 131:205–13. 10.1016/j.jprot.2015.11.003 26546555

[24] Silva EM Conde JN Allonso D Nogueira ML Mohana-Borges R. Mapping the interactions of dengue virus NS1 protein with human liver proteins using a yeast two-hybrid system: identification of C1q as an interacting partner. PLoS ONE (2013) 8:e57514. 10.1371/journal.pone.0057514 23516407 PMC3597719

[25] Ochsenbein AF Fehr T Lutz C Suter M Brombacher F Hengartner H. Control of early viral and bacterial distribution and disease by natural antibodies. Science (1999) 286:2156–9. 10.1126/science.286.5447.2156 10591647

[26] Ehrenstein MR Notley CA. The importance of natural IgM: scavenger, protector and regulator. Nat Rev Immunol. (2010) 10:778–86. 10.1038/nri2849 20948548

[27] Beebe DP Cooper NR. Neutralization of vesicular stomatitis virus (VSV) by human complement requires a natural IgM antibody present in human serum. J Immunol. (1981) 126:1562–8. 6259260

[28] Tesfay MZ Ammayappan A Federspiel MJ Barber GN Stojdl D Peng KW. Vesiculovirus neutralization by natural IgM and complement. J Virol. (2014) 88:6148–57. 10.1128/JVI.00074-14 24648451 PMC4093862

[29] Seiler P Kalinke U Rulicke T Bucher EM Bose C Zinkernagel RM. Enhanced virus clearance by early inducible lymphocytic choriomeningitis virus-neutralizing antibodies in immunoglobulin-transgenic mice. J Virol. (1998) 72:2253–8. 9499083 10.1128/jvi.72.3.2253-2258.1998 PMC109522

[30] Baumgarth N Herman OC Jager GC Brown L Herzenberg LA Herzenberg LA. Innate and acquired humoral immunities to influenza virus are mediated by distinct arms of the immune system. Proc Natl Acad Sci USA. (1999) 96:2250–5. 10.1073/pnas.96.5.2250 10051627 PMC26769

[31] Baumgarth N Herman OC Jager GC Brown LE Herzenberg LA Chen J. B-1 and B-2 cell-derived immunoglobulin M antibodies are nonredundant components of the protective response to influenza virus infection. J Exp Med. (2000) 192:271–80. 10.1084/jem.192.2.271 10899913 PMC2193249

[32] Choi YS Baumgarth N. Dual role for B-1a cells in immunity to influenza virus infection. J Exp Med. (2008) 205:3053–64. 10.1084/jem.20080979 19075288 PMC2605232

[33] Diamond MS Sitati EM Friend LD Higgs S Shrestha B Engle M. A critical role for induced IgM in the protection against West Nile virus infection. J Exp Med. (2003) 198:1853–62. 10.1084/jem.20031223 14662909 PMC2194144

[34] Mehlhop E Whitby K Oliphant T Marri A Engle M Diamond MS. Complement activation is required for induction of a protective antibody response against West Nile virus infection. J Virol. (2005) 79:7466–77. 10.1128/JVI.79.12.7466-7477.2005 15919902 PMC1143684

[35] Mehlhop E Diamond MS. Protective immune responses against West Nile virus are primed by distinct complement activation pathways. J Exp Med. (2006) 203:1371–81. 10.1084/jem.20052388 16651386 PMC2121216

[36] Fuchs A Pinto AK Schwaeble WJ Diamond MS. The lectin pathway of complement activation contributes to protection from West Nile virus infection. Virology (2011) 412:101–9. 10.1016/j.virol.2011.01.003 21269656 PMC3057364

[37] Mehlhop E Fuchs A Engle M Diamond MS. Complement modulates pathogenesis and antibody-dependent neutralization of West Nile virus infection through a C5-independent mechanism. Virology (2009) 393:11–5. 10.1016/j.virol.2009.08.019 19744691 PMC2753729

[38] Vasantha S Coelingh KL Murphy BR Dourmashkin RR Hammer CH Frank MM. Interactions of a nonneutralizing IgM antibody and complement in parainfluenza virus neutralization. Virology (1988) 167:433–41. 10.1016/0042-6822(88)90105-5 2849234

[39] Spear GT Sullivan BL Landay AL Lint TF. Neutralization of human immunodeficiency virus type 1 by complement occurs by viral lysis. J Virol. (1990) 64:5869–73. 1700828 10.1128/jvi.64.12.5869-5873.1990 PMC248749

[40] Spear GT Takefman DM Sullivan BL Landay AL Zolla-Pazner S. Complement activation by human monoclonal antibodies to human immunodeficiency virus. J Virol. (1993) 67:53–9. 7677959 10.1128/jvi.67.1.53-59.1993 PMC237336

