# Supplementary material for: Active Human Complement Reduces the Zika Virus Load via Formation of the Membrane-Attack Complex
Source: Front Immunol. 2018 Oct 17;9:2177. doi: 10.3389/fimmu.2018.02177 (PMC6199351; doi:10.3389/fimmu.2018.02177)

**Supplementary data**

**Suppl. Figure 1: Absence of flavivirus-specific antibodies in the serum pool used for neutralization of ZIKV**

Since we were interested in complement-mediated neutralization of ZIKV virions, the purchased serum pool was analyzed by ELISA for the entire spectrum of flavivirus-IgG antibodies in order to ensure that no cross-reactive immunoglobulins were present**.** To this end**,** recombinant purified flavivirus E proteins from DENV serotypes 1 and 2, ZIKV, YFV, WNV and TBEV containing a strep-tag were bound to Streptactin-coated plates (Iba GmbH, Germany). Serial dilutions of the human serum pool (and positive controls, not shown) were added. Bound antibodies were detected with Goat Anti-Human IgG (H+L) Cross Adsorbed Secondary Antibody-HRP (Thermo Pierce). The cut-off value (dotted line) was calculated as the mean of six negative serum samples plus three standard deviations.

Flavivirus-specific antibodies were not detected, confirming the absence of pre-existing flavivirus infections or immunizations (e.g. vaccinations against yellow fever virus) of serum donors.


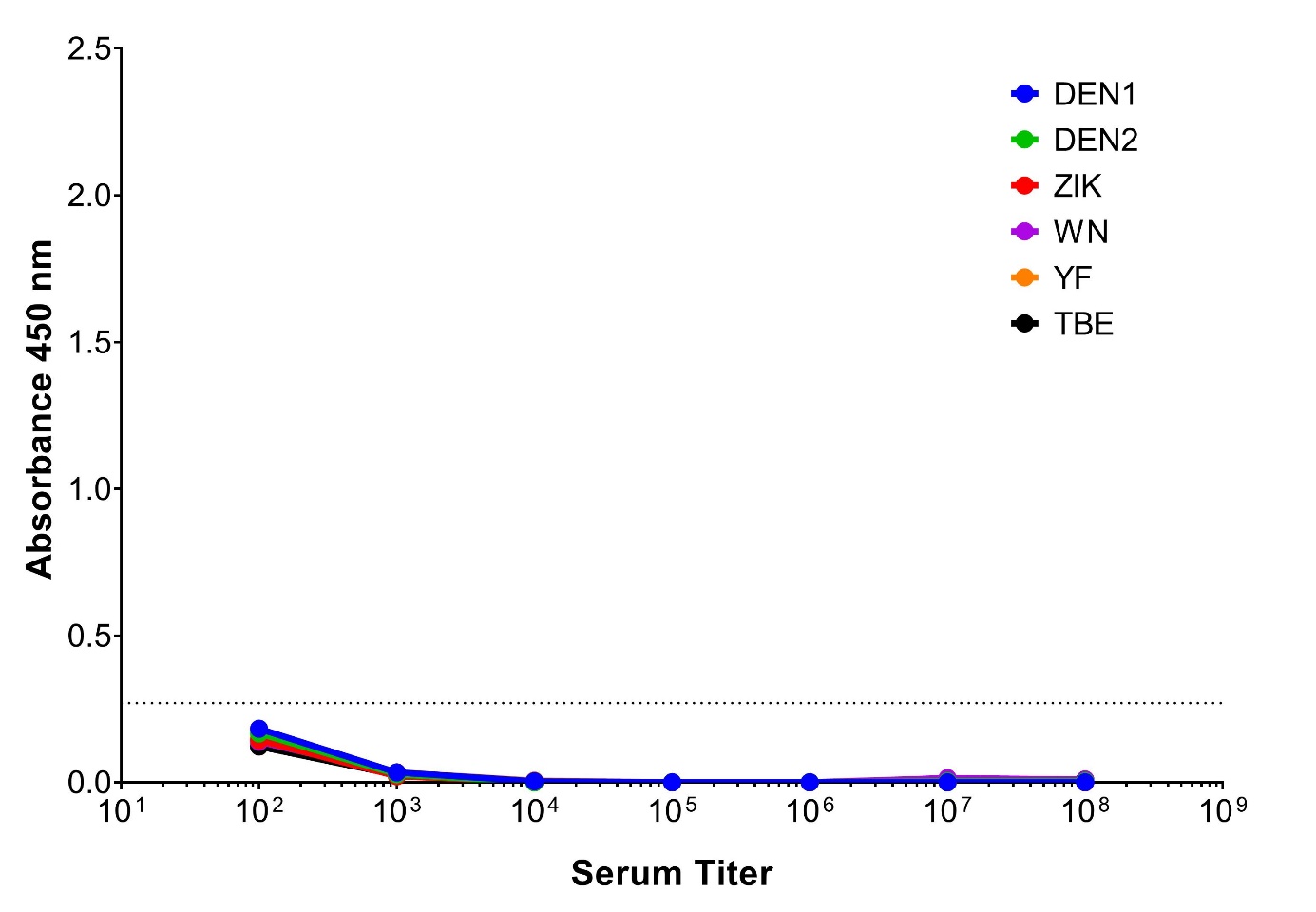


**Suppl. Figure 2. Detection of insect cell-specific IgMs in hiNHS**

Based on our experimental data, we tested the presence of IgM which recognized insect cell-specific compounds in our serum pool. To avoid lysis of the insect cells, the serum pool was heat inactivated and incubated with the cells at a 1:10 dilution. As a control, the human cell line A549 was used (left histogram). After washing, cells were incubated with allophycocyanin (APC)-labeled anti-human IgM or a control isotype. Bound IgM was detected by FACS analysis. The clear shift of the IgM signal indicated the presence of insect cell-specific IgM (right histogram).


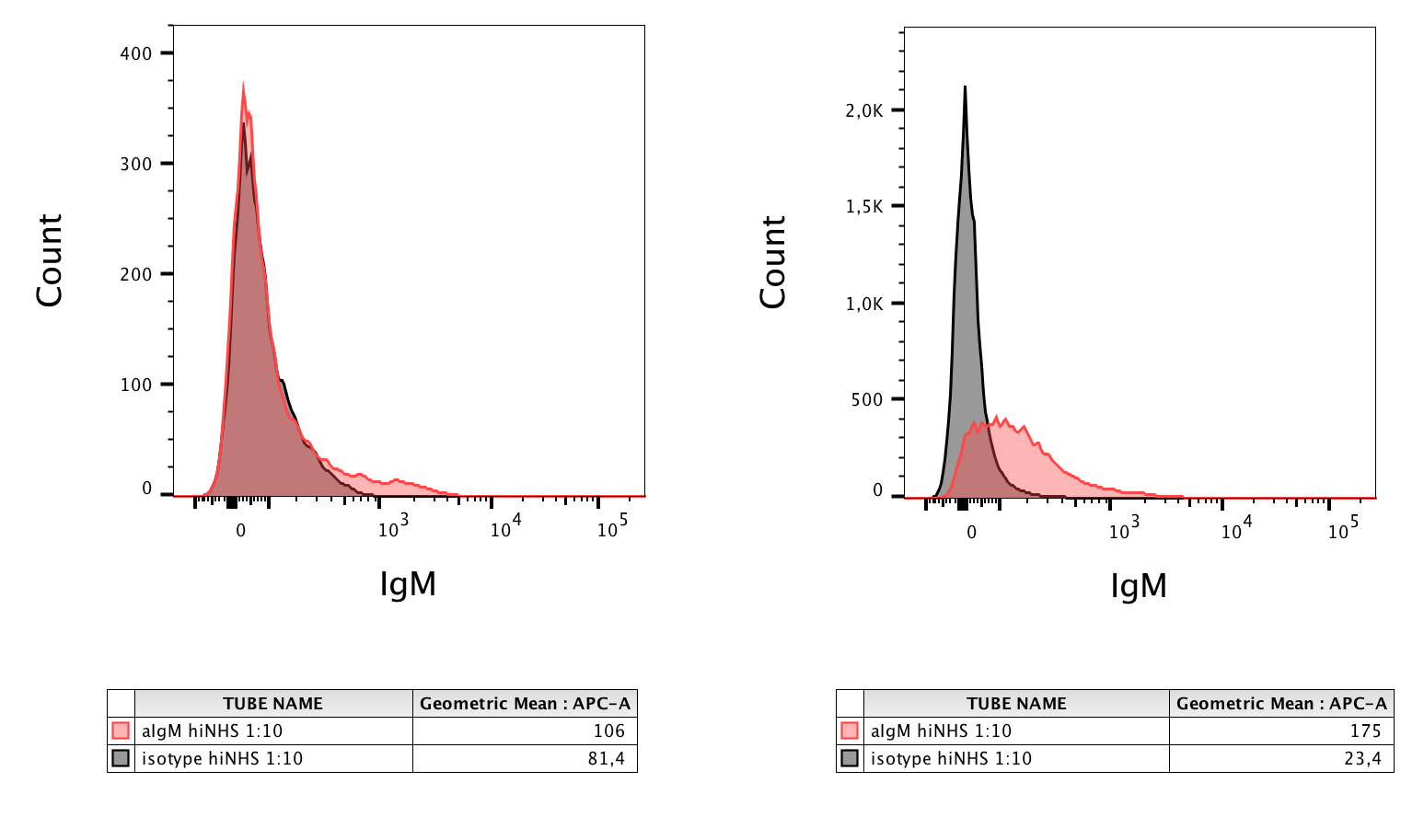

Supplement: Supplementary file 1 [file Data_Sheet_1.docx]
